# Role of cancer-associated fibroblasts in the progression, therapeutic resistance and targeted therapy of oesophageal squamous cell carcinoma

**DOI:** 10.3389/fonc.2023.1257266

**Published:** 2023-10-20

**Authors:** Mengying Xue, Yusuo Tong, Yaozu Xiong, Changhua Yu

**Affiliations:** Department of Radiotherapy, The Affiliated Huaian No.1 People’s Hospital of Nanjing Medical University, Huaian, China

**Keywords:** cancer-associated fibroblasts, oesophageal squamous cell carcinoma, tumour microenvironment, tumour progression, treatment resistance

## Abstract

Oesophageal squamous cell carcinoma (ESCC) is one of the most aggressive malignant tumours with high morbidity and mortality. Although surgery, radiotherapy and chemotherapy are common treatment options available for oesophageal cancer, the 5-year survival rate remains low after treatment. On the one hand, many oesophageal cancers are are discovered at an advanced stage and, on the other hand, treatment resistance is a major obstacle to treating locally advanced ESCC. Cancer-associated fibroblasts (CAFs), the main type of stromal cell in the tumour microenvironment, enhance tumour progression and treatment resistance and have emerged as a major focus of study on targeted therapy of oesophageal cancer.With the aim of providing potential, prospective targets for improving therapeutic efficacy, this review summarises the origin and activation of CAFs and their specific role in regulating tumour progression and treatment resistance in ESCC. We also emphasize the clinical potential and emerging trends of ESCC CAFs-targeted treatments.

## 1 Introduction

Oesophageal cancer (EC) is one of the most prevalent gastrointestinal malignant tumours, ranking eighth in worldwide tumour incidence and sixth in death (1). It is categorised into two mainr histological subtypes: oesophageal squamous cell carcinoma (ESCC) and oesophageal adenocarcinoma (EAC) (2).The most prevalent kind of EC, that take account over 90% of all EC occurrences, is ESCC. Because the early symptoms are not evident, over 70% ESCC patients are detected at the stage of advanced tumour, which results in poor 5-year survival rates (3). Early EC can be treated via endoscopic resection and radical oesophagectomy (4). Treatment options for locally advanced ESCC include radical oesophagectomy and radical synchronous chemo-radiotherapy (CRT) (5). In addition, patients with locally advanced EC require multidisciplinary treatment (6), such as simultaneous neoadjuvant CRT to shrink the tumour followed by surgical resection (7). Overall, the treatment of EC requires the close cooperation of multiple treatment strategies. Despite progressive advancements in the treatment of EC, therapeutic efficacy and prognosis remain poor among most patients with advanced EC owing to EC development and treatment resistance. Moreover, these patients have a 5-year survival rate of less than 20% (8). Therefore, understanding of EC is necessary for developing or optimising treatment strategies to enhance patients’ quality of life.

The tumour microenvironment (TME) refers to the environment surrounding tumour cells. It mainly includes peripheral blood vessels, stromal cells (including cancer-associated fibroblasts [CAFs] and endothelial cells), immune cells, non-cellular components (such as cytokines and growth factors), hormones and the extracellular matrix(ECM) (9–12). CAFs are an essential part of TME and have different sources and phenotypes that regulate cancer progression and treatment response (13). In addition, CAFs engage in strong crosstalk with cancer cells and involve in a number of biological processes, including wound healing, inflammation, tumour initiation, tumour progression and immune rejection, which may lead to treatment failure, especially resistance to chemotherapy and radiation therapy (14, 15). Consequently, CAFs are crucial in fostering the growth of tumours. Cancer cells can induce the transformation of normal fibroblasts (NFs) to CAFs in order to foster the development of an immunosuppressive and pro-survival microenvironment (16). Owing to these characteristics, CAFs are the primary stromal cells affecting tumour growth and are excellent targets for the treatment of tumours (17).

Elucidating the mechanisms underlying EC progression, propagation and treatment resistance and exploring therapeutic strategies targeting CAFs are promising approaches to improving treatment outcomes in EC. Therefore, this review aimed to contribute to the existing knowledge on CAFs and summarise potential therapeutic strategies that focus on CAFs for EC treatment.

## 2 Origins and activation of CAFs

**Figure 1:**
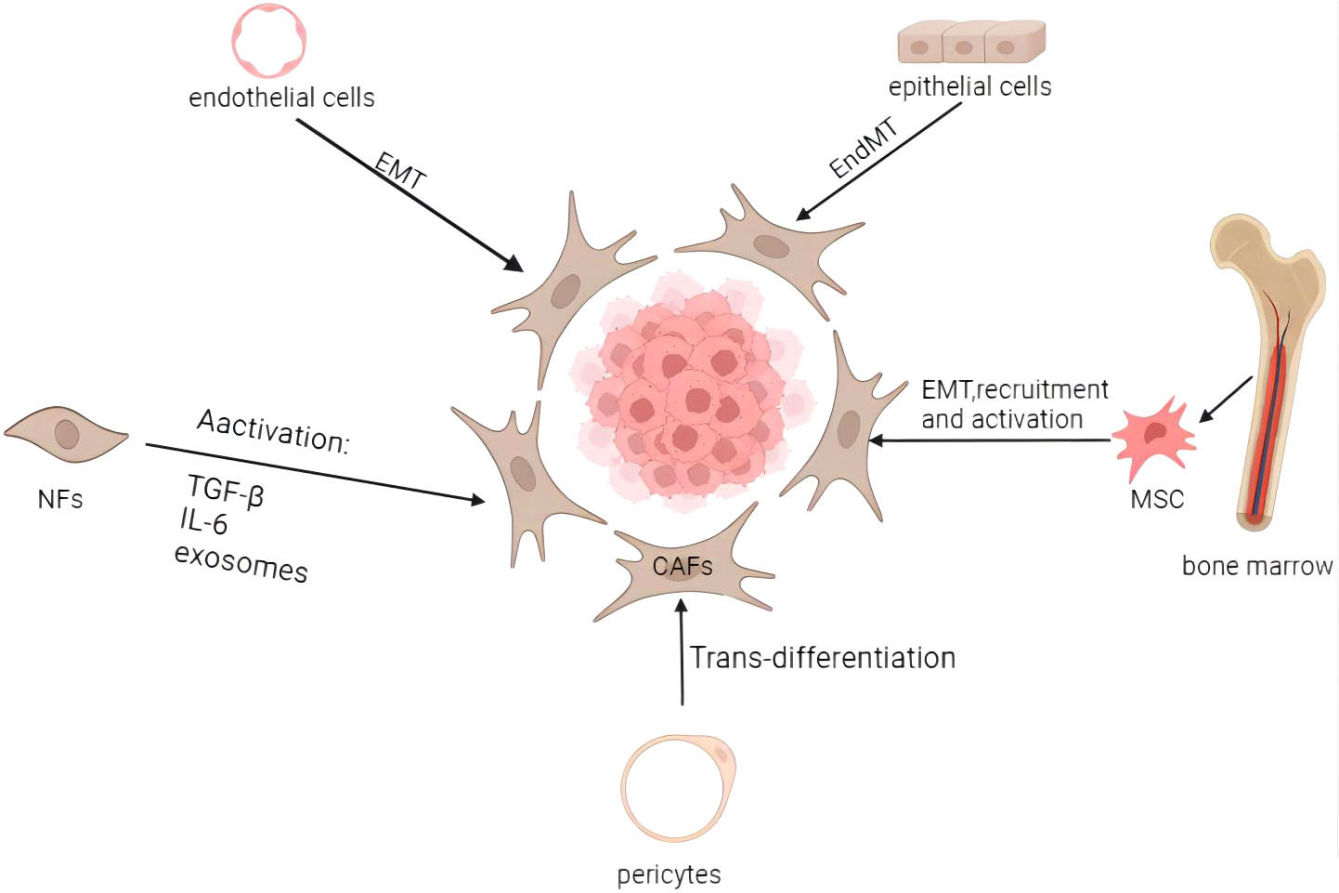
Origins and activation of cancer-associated fibroblasts. CAFs may come from a variety of biological origins, including bone marrow MSCs (via EMT, recruitment, and activation), normal fibroblasts (through activation), epithelial cells (through EMT), endothelial cells (through EndMT), and pericytes (through trans-differentiation), among others. Activation of dormant fibroblasts can be induced by inflammatory cytokines and other factors, as well as by a number of other routes. Image created with BioRender.com.

Numerous studies have demonstrated the heterogeneity and complexity of the CAF population. It’s plausible that the CAFs’ heterogeneity results from their several potential cellular ancestries. CAFs develop from various progenitors, including NFs, epithelial and endothelial cells, pericytes and bone marrow-derived cells (**Figure 1**) (18). CAFs may develop from NFs that have been stimulated by tumor cells in the area. Mesenchymal stem cells (MSCs) from bone marrow can also develop into CAFs in addition to natural sources (19). CAFs can also originate from epithelial cells via epithelial-mesenchymal transition(EMT) (20) and from endothelial cells via endothelial-mesenchymal transition(EndMT) (21). Pericytes and other transdifferentiated cells are less frequent progenitors of CAFs (22). The diversity of CAF sources contributes to the development of diverse CAF subtypes expressing various markers (23). These subtypes perform distinct activities and are associated with the classification of EC, thereby influencing clinical symptoms and prognosis.

CAFs stimulate tumour formation, and tumour cells produce CAFs. CAFs interact with tumour cells to create a microenvironment that promotes tumour progression, invasion, metastasis and treatment resistance. Numerous researches have revealed that cancer cells can transform NFs to activated CAFs. Some fibroblast activators, including tumour growth factor-beta (TGF-β) and interleukin-6 (IL-6), are involved in cellular signalling pathways through the corresponding receptors, and their expression is linked to the formation of CAF subtypes with improved synthetic and secretory capabilities.

TGF-β is a well-known fibroblast activator. Fang et al. (24) analysed sequencing data extracted from The Cancer Genome Atlas and RNA microarray data (GSE53625) and stimulated ESCC cell lines with or without TGFβ1. They found that the excessive expression of laminin subunit gamma 1 (LAMC1) in ESCC cells affects the outcome of patients. TGF-β1 can increase the expression of LAMC1 by activating SMAD family member 4 (SMAD4) and SP1. LAMC1 promotes the formation of inflammatory CAFs (iCAFs) by the CXCR2–PSTAT3 axis and increases CXCL1 secretion. iCAFs promote tumour growth both *in vivo* and vitro. IL-6 facilitates the interaction between tumour cells and CAFs by enhancing fibroblast activation and tumour cell proliferation. Besides, Chen et al. (25) demonstrated that expression of Annexin A1 (ANXA1) produced by normal oesophageal epithelial cells acts as a ligand molecule that can control and maintain NF homeostasis by interacting with the receptor formyl peptide receptor type 2 (FPR2) on fibroblasts. As a result of reduced ANXA1-FPR2 signalling between precancerous/malignant epithelial cells and fibroblasts, the promotion of the conversion of NFs to CAFs was enhanced by increased TGF-β production in ESCC TME.

Karakasheva et al. (26) reported that IL-6 expression was upregulated in co-cultured ESCC cells and CAFs. Chronic inflammation leads to NFs to activate and transform into CAFs. CAFs not only secrete high levels of IL-6 but also promote IL-6 secretion from tumour cells. IL-6 activates the corresponding receptor IL-6R on tumour cells and CAFs by means of autocrine–paracrine, which leads to differential activation of the STAT3 and MEK/ERK signalling pathways, resulting in tumour development.

Exosomes secreted by tumour cells may contain functional DNA fragments and coding and non-coding RNAs and other components. Additionally, they can deliver active growth factors and cytokines to induce fibroblasts’ activation and differentiation. Tong et al. (27) reported that lncRNA POU3F3 was transported from ESCC cells to NFs by exosomes and regulated the activation of fibroblasts. Activated fibroblasts further enhanced the progression of ESCC cells by secreting IL-6. These findings suggest that exosomes secreted by ESCC cells can improve the activation of NFs to CAFs.

Fang et al. (28) found that urokinase-type plasminogen activator (PLAU) secreted by ESCC cells contributed the transformation of fibroblasts to iCAFs and improved the expression and secretion of IL-8 through the urokinase-type plasminogen activator receptor (uPAR)–Akt–NF-κB pathway. Conversely, IL-8 released by CAFs promoted the upregulation of PLAU in tumour cells, thereby accelerating the development of ESCC.

NADPH oxidase 5 (NOX5) is overexpressed in the tumour tissues of ESCC patients, and this overexpression has negative association with the development and prognosis of ESCC. Chen et al. (29) demonstrated that NOX5 triggered intra-tumoural Src/nuclear factor-B signalling to promote the secretion of tumour necrosis factor- alpha (TNF-α), IL-1β and lactate in tumour cells. Additionally, these tumour cells altered the cytokine of activated CAFs, which further boosted the activation of NFs or mesenchymal stem cells (MSCs) to CAFs and stimulated lymphangiogenesis to promote ESCC progression. Consequently, TNF-α, IL-1β and lactate stimulated CAF activation and promoted the production of IL-6, IL-7, IL-8, CCL5 and TGF-β1 in CAFs, which in turn promoted the growth of ESCC cells that were positive for NOX5. Therefore, cytokine networks can promote tumour growth by facilitating communication between ESCC cells and associated stromal cells.

ESCC cells is significant in the development of CAFs. They can stimulate the transformation of NFs to CAFs through specific mechanisms. Activated CAFs can adapt to tumour cells and co-evolve with them to support the development, multiplication, infiltration and metastasis of ESCC cells through various signalling pathways, including paracrine signalling.

## 3 Mechanisms of CAFs in the progression of ESCC

CAFs have a higher metabolic activity and stronger proliferative ability than NFs. Many tumour signalling pathways are abnormally activated in CAFs to enhance the progression and spread of tumours. This section describes the biological behaviour of CAFs and the mechanisms through which they control tumour growth. CAFs can not only directly affect tumour cells through paracrine signalling but also indirectly control immune processes for regulating immune evasion or metabolism for tumour growth (13) (**Table 1**).

**Table 1:** Mechanisms through which CAFs promote biological processes in ESCC.

Perspectives	Mediators	Mechanisms	References
To enhance tumour progression, proliferation and metastasis	TGF-β	Upregulates LAMC1 by activating SP1 and SMAD4	Fang et al. (24)
	HIC-5	Regulates cytokine production and alters the ECM	Du et al. (30)
	NOX 5	Promotes the production of TNF-α, IL-1β and lactate by activating Src/NF-κB signalling	Chen et al. (29)
	MT2A	Promotes IGFBP2 production and secretion through the NF-κB, AKT and ERK signalling pathways	Shimizu et al. (31)
	POSTN	Stimulates ADAM17 activity by activating the integrin αvβ3 or the αvβ5–ERK1/2 pathway	Ishibashi et al. (32)
	CCL5	Promotes the growth of ESCC cells both *in vitro* and *in vivo* through ERK1/2 signaling	Dunbar et al. (33)
	PLAU	Increases the expression of IL-8 via the uPAR/Akt/NF-κB pathway	Fang et al. (28)
	uPA	Enhances ESCC cell growth, proliferation, migration and invasion by the PI3K/Akt and ERK signalling pathways	Tian et al. (34)
	PAI-1	Induces cell migration and invasion through LDL receptor-associated protein 1	Sakamot et al. (35)
	lncRNA POU3F3	Transforms NFs to CAFs through exosomes and stimulates the proliferation and cisplatin resistance in ESCC cells via the production of IL-6	Tong et al. (27)
	miR-3656	Activates the PI3K/Akt and β-catenin signalling pathways by regulating ACAP2 downregulation	Jin et al. (36)
	LINC01410	Promotes EMT by upregulating miR-122-5P and increasing the expression of PKM2	Shi et al. (37)
	mir-100-5p	Stimulates lymphangiogenesis through IGF1R/PI3K/AKT axis and aids in the spread of tumor lymph nodes	Chen et al. (38)
	exosomal proteins	boostes oesophageal cancer cells’ ability to proliferate, invade, and migrate	Wang et al. (39)
	Sonic Hedgehog	Enhances the growth and migration of oesophageal cancer cells	Zhao et al. (40)
To evade immune surveillance	PD-1/PD-L1	Facilitates tumour cell survival by helping the cells to evade recognition by T cells	Qiu et al. and Higashino et al. (41, 42)
	M2 macrophages	Suppresses antitumour immune responses and secrets anti-inflammatory molecules including TGF-β, IL10 and Arginase1 as well as PD-L1 and PD-L2	Sakamoto et al. (35)
	IDO	Promotes immune escape by inducing apoptosis of T and NK cells and enhancing Treg activity	Cui et al. (43)
	M-MDSCs	Induces paracrine and autocrine functions of IL-6 to activate STAT3 signalling	Zhao et al. (44)
	TILs	CAFs form an immune microenvironment by promoting the migratory and invasive capabilities of FoxP3 TILs while suppressing the infiltration of CD8 TILs	Kato et al. (45)
	WNT2	Restores antitumour T cell responses and improves the efficacy of anti-PD-1 treatment	Huang et al. (46)
	FGF2 and SPRY1	Impairs T cell activity and promotes ESCC progression	Chen et al. (47)
To participate in metabolism	Reverse Warburg effect	Some epithelial cancer cells metabolically supported the growth of adjacent stromal fibroblasts	Du et al. (30)
		The presence of pro-inflammatory cytokines can stimulate glycolysis, which may lead to the local accumulation of energy-rich metabolites	(48–50)

CAFs secrete various factors, including TGF-β, IL-6 and insulin-like growth factor-binding protein 2 (IGFBP2). They can promote tumour angiogenesis, tumour cell proliferation and tumour invasion by influencing several signalling pathways in the TME (51).

TGF-β can induce tumour initiation and immune escape by regulating cancer cell–ECM interactions (52). Fang et al. (24) reported that TGF-β1 upregulated LAMC1 by activating SP1 and SMAD4 in a synergistic manner. Through Akt–NF-κB–MMP-9/14 signalling, LAMC1 promoted the multiplication, infiltration of tumour cells, increased CXCL1 production and enhanced the development of iCAFs, resulting in enhanced tumour growth both *in vitro* and *in vivo*. Furthermore, Du et al. (30) reported that hydrogen peroxide-inducible clone 5 (HIC-5) was substantially expressed in CAFs isolated from the tumour stroma of patients with ESCC. Knockout of HIC-5 in CAFs restrained the migratory and invasive capabilities of ESCC cells *in vitro*. Mechanistically, HIC-5 enhances the migratory and invasive capabilities of ESCC cells through regulating cytokine production and altering the ECM. It is linked to positive lymph node metastasis and increased TNM stages. It controls cell migration by regulating cell motility and the Rho GTPase signalling pathway through TGF-β/Smad signalling. Upregulated IL-6 can boost the proliferation of CAFs and ESCC cells by upregulating PSTAT3 and PERK 1/2 expression and downregulating caspase-3 cleavage, resulting in decreased apoptosis and increased tumour growth and invasion. According to Chen et al. (29), NOX5 increased ESCC cell production of TNF-, IL-1, and lactate via triggering Src/NF-B signaling. In addition, NOX5 stimulated CAFs to secrete IL-6, IL-7, IL-8, CCL5, and TGF-1. The NOX5-positive ESCC cells’ malignant behavior was sped up by these CAF-secreted cytokines. These CAFs-secreted cytokines accelerated the pernicious behaviour of NOX5-positive ESCC cells. Shimizu et al. (31) reported that metallothionein 2A (MT2A) was substantially expressed in CAF-like cells. By the NF-B, AKT, and ERK signaling pathways, MT2A boosts the synthesis and release of IGFBP2 in CAF-like cells and encourages the migratory and invasive properties of ESCC cells.Additionally, MT2A takes part in tumour cell expansion, invasion and migration in ESCC. CAFs accelerate the development of tumours by secreting TGF-β and IL-6. Ishibashi et al. (32) demonstrated that periostin (POSTN) was significantly expressed by CAFs in ESCC tissues and that the CAF-cultured medium substantially enhanced the migratory, invasive, proliferative and colony formtion capabilities of ESCC cells in a POSTN-dependent way. CAF-derived POSTN stimulates the activity of a disintegrin and metalloproteinase 17 (ADAM17) by activating the integrin αvβ3 or αvβ5–ERK1/2 pathway, thus contributing to the growth, proliferation, invasion and metastasis of ESCC. By using unbiased cytokine arrays, Dunbar et al. (33) discovered CC motif chemokine ligand 5 (CCL5) that is elevated during co-culture of ESCC cells and CAFs. Lack of CCL5 produced by tumor cells prevented the growth of ESCC cells both *in vitro* and *in vivo* through lowering ERK1/2 signaling. Additionally, it decreases the percentage of CAFs *in vivo* recruited by xenograft tumors. CCL5 is a ligand for the CC motif receptor 5 (CCR5), for which maraviroc has received clinical approval. The use of maraviroc decreases tumor volume, CAF recruitment, and ERK1/2 signaling. Low-grade oesophageal cancer has a bad prognosis when CCL5 or CCR5 expression is high.

The PLAU system includes PLAU, uPAR and plasminogen activator inhibitor-1 (PAI-1) (53). Through a proteolytic pathway, intracellular signalling and chemokine activation, this system regulates cell adhesion, growth, proliferation, invasion, metastasis and other functions (54). PLAU which is overexpressed in numerous tumours serves as essential for the growth and spread of tumours. According to Fang et al. (28), PLAU stimulated the expression of fibroblasts to iCAFs and increased the conversion of IL-8 by the uPAR/Akt/NF-κB pathway. In addition, IL-8 produced by CAFs stimulated the increased reflection of PLAU in tumour cells, thus accelerating the development of ESCC. Tian et al. (34) used an antibody array and identified uPA as one primary protein with high secretion in CAFs in contrast to NFs. uPA was primarily distributed in the boundary of tumour and stromal tissues, tumour nest stroma and tumour body. Elevated interstitial uPA levels were related to tumour infiltration and overall survival(OS) in ESCC patients. Through the PI3K/Akt and ERK pathways, uPA promoted ESCC cell progression and metastasis. Sakamoto et al. (35) found that CAF-like cell-derived PAI-1 promoted the migration and invasion of ESCC cells and macrophages through LDL receptor-associated protein 1 (LRP1), a receptor of PAI-1.

Exosomes are small extracellular vesicles that play a crucial role in intercellular communication. They can transfer various biomolecules for example lipids, proteins and messenger and non-coding RNAs, which can induce the activation of CAFs and promote cell proliferation, invasion, migration and survival (55). Exosomes are found in different body fluids and act as important mediators of the crosstalk of CAFs with tumour cells (56). As mentioned earlier, exogenous lncRNA POU3F3 secreted by ESCC cells can trigger NFs to CAFs through exosomes, thereby mediating fibroblast activation (27). Activated fibroblasts can stimulate the progressive, migratory and invasive capabilities of ESCC cells by releasing IL-6. Jin et al. (36) reported that exosomes secreted by CAFs possess a high concentration of HSA-miR-3656 mRNA, which enhances the proliferative, migratory and invasive characteristics of ESCC cells. Exosomal miR-3656 stimulates the PI3K/Akt and β-catenin signalling transductions by suppressing ACAP2, thereby promoting cancer progression. Except microRNAs (miRNAs), exosomal lncRNAs can contribute to CAF-mediated promotion of invasive and migratory capabilities of ESCC cells. For instance, LINC01410 triggered by CAFs-Exo promotes EMT in ESCC by upregulating miR-122-5P and boosting the production of pyruvate kinase M2, which leads to tumour metastasis (37). Otherwise, Chen et al. (38) found that mir-100-5p is produced by CAF and is present in exosomes. It stimulates lymphangiogenesis through IGF1R/PI3K/AKT axis and aids in the spread of tumor lymph nodes. According to Wang et al. (39), CAF-derived exosomes greatly boosted oesophageal cancer cells’ ability to proliferate, invade, and migrate. Additionally, they created a risk profile using exosomal proteins generated from CAF and discovered that the prognosis for the high-risk group was much poorer, indicating that exosomal proteins produced from CAF may be used as an independent prognostic indicator. They also noticed a substantial relationship in the study population between risk ratings and immune cell infiltration, immunotherapy response, and chemotherapy sensitivity.

The Hedgehog signalling pathway is crucial in controlling differentiation and proliferation of tumours during vertebrate embryogenesis, and its involvement has also been observed in several cancers, including ESCC. Zhao et al. (40) reported that Sonic Hedgehog is strongly produced in CAFs lysis solution, conditioned medium of cultured CAFs and exosomes generated from CAFs. Cyclopamine’s inhibition of the Hedgehog signaling pathway reduced the tumour-promoting capacity of CAFs. These findings imply that exosomes derived from CAFs elevate the proliferation and metastasis of ESCC cells via the Sonic Hedgehog pathway. By secreting exosomes, CAFs can regulate tumour cells’ behavior. However, tumour cells can modify NFs by secreting exosomes that deliver cytokines and activate pathways to create a favourable microenvironment for their survival and progression.

CAFs significantly influence the immunological milieu of cancer tissues primarily by promoting tumourigenesis by inducing chronic swelling and reducing the immune system’s response to tumours (57). TME consists of numerous immune cell types, such as antitumour cytotoxic T cells, regulatory T cells (Tregs), natural killer (NK) cells, dendritic cells, M1 and M2 macrophages and immunosuppressive myeloid-derived suppressor cells (MDSCs). CAFs have a tight relationship with immune and tumour cells. Resident fibroblasts surrounding tumour cells are usually the major source of CAFs, which can be induced by various growth factors secreted by tumour cells, such as TGF-β (58). In addition, CAFs can communicate with immune cells that have infiltrated the tumor within the TME by releasing a variety of cytokines, growth factors and other relative molecules, thereby producing an growth-promoting and immunosuppressive TME that allows cancer cells to evade immune surveillance (16). Therefore, CAFs take a significant part in the formation of the immune microenvironment (59).

Immunosuppressive cells may be attracted by and differentiated by CAFs. The PD-1/PD-L1 pathway promotes tumour cell survival by helping the cells to evade T cell recognition. Qiu et al. (41) found that PD-L1 expression was upregulated in CAFs in ESCC. The proliferation and migration of ESCC cells and peripheral blood monocyte-derived macrophage-like cells were both boosted by CAF-like cells, according to an indirect co-culture experiment involving human bone marrow-derived MSCs and ESCC cells. In addition, CAF-like cells triggered M2 polarisation, and macrophage-like cells were actively involved in suppressing antitumour immune responses (42). Monocytes are attracted by CAFs, which then induce them to acquire an immunosuppressive phenotype. They allow M2 macrophages to provide an immunosuppressive microenvironment conducive to tumour progression by triggering anti-inflammatory molecules including TGF-β, IL-10, Arginase1 and PD-L1 and PD-L2 (35). The immunosuppressive factor indoleamine Indoleamine 2,3-dioxygenase (IDO) (43) promotes immune escape by inducing apoptosis of T and NK cells and increasing Treg activity. IDO is manifested by CAFs and endothelial cells in the tumor stroma of ESCC, which raises the possibility that ESCC cells can avoid immune surveillance by way of CAFs that express IDO. MDSCs are heterogeneous cells that inhibit immune responses, including the effector functions of T cells and NK cells, under pathological conditions (60, 61). Zhao et al. (44) found that co-activation of STAT3 signalling by the paracrine and autocrine actions of CAF-derived IL-6 increased the production of mononuclear myeloid-derived suppressor cells (M-MDSCs), which promoted tumour proliferation and chemotherapy resistance by suppressing immune responses. 140 esophageal cancer cases in total were examined by IHC from Kato et al (45) for CAFs and CD8(+) or forkhead box protein 3 (FoxP3(+)) TILs. CAFs were found to have a negative relationship with CD8 TILs and have a positive relationship with FoxP3 TILs in intra-tumour tissues. Consequently, CAFs could create an immune microenvironment by promoting the migratory and invasive capabilities of FoxP3 TILs while preventing CD8 TILs’ infiltration. Correspondingly, Huang et al. (46) demonstrated a negative relationship between WNT2 CAFs and activated CD8 T cells in basic ESCC cells. In addition, in mice ESCC and colorectal cancer homologous tumor models, the anti-WNT2 monoclonal antibody greatly restored anti-tumour T-cell responses and enhanced the therapeutic effect of anti-PD-1 treatment. Chen et al. (47) demonstrated a strong correlation between the expression of FGF2 and Sprouty RTK signalling antagonist 1 (SPRY1) in EC. FGF2 overexpression in CAFs dramatically increased SPRY1 expression, impaired T cell activity and accelerated ESCC progression, suggesting that CAFs are crucial in the development of an immunosuppressive TME (62).

Overall, CAFs is essential in creating an immunosuppressive TME through various mechanisms, including recruitment of immunosuppressive cells, induction of differentiation and interaction with immune cells. Therefore, novel immunotherapies targeting CAFs may be beneficial for the treatment of ESCC.

CAFs are involved in metabolic processes in ESCC through numerous factors, such as cytokines, metabolites, and extracellular vesicles (63). When nutrients are insufficient to support tumorigenesis in the initial stages of tumor growth, CAFs use the tricarboxylic acid cycle (TCA) to produce energy to support biological functions. The’reverse Warburg effect’ is a term used to describe this phenomenon (64).

Du et al. (30) altered several metabolic pathways based on RNA-seq analysis of comparative HIC-5-knockdown of CAFs verus control CAFs. Some epithelial cancer cells metabolically supported the growth of adjacent stromal fibroblasts (reverse Warburg effect). Its data indicated that several metabolic pathways including FoxO and AMPK signalling pathways were altered by silencing HIC-5 in CAFs, suggesting that HIC-5 participates in communication between CAFs and cancer cells by affecting metabolite synthesis. CAFs differentiate into myofibroblasts and aerobic glycolysis is used by CAFs to create lactate and pyruvate. These energy-rich metabolites are absorbed by cancer cells and used for the TCA cycle in the mitochondria. Efficient ATP synthesis creates an appropriate environment for cancer cells to multiply. Pro-inflammatory cytokines can accelerate glycolysis (48–50) and CAF-derived HIC-5 increases the release of cytokines like CCL2, which may cause the local accumulation of energy-rich metabolites Altogether, CAF-derived HIC-5 can accelerate cancer growth by modulating metabolic signalling pathways, promoting inflammatory states and upregulating glycolysis, thus providing metabolites to cancer cells. However, the mechanisms through which metabolic processes contribute to the regulation of EC cell behaviour remain elusive, and extensive investigation is warranted to develop novel therapeutic strategies targeting metabolic processes for inhibiting the malignant evolution and proliferation of EC cells. Therefore, metabolomics is a promising approach to developing novel therapeutic strategies for ESCC.

## 4 Role of CAFs in treatment resistance in ESCC

ESCC is a highly aggressive malignant tumour with a high recurrence rate and a low 5-year OS rate (<15%) owing to drug resistance (8). Therefore, identifying effective therapeutic targets is necessary for overcoming drug resistance and improving the survival quality and prognosis of patients with advanced ESCC.

Recent studies have demonstrated that numerous factors in the TME can contribute to drug resistance. Cytokines are widely found in TME and play a key role in providing a favourable environment for tumour progression. In addition to cytokines, chemokines and their receptors greatly expressed in multiple malignancies are associated with tumour proliferation, migration, invasion and metastasis. Zhang et al. (65) demonstrated that CAFs significantly increased the drug resistance of ESCC cells by secreting TGF-β1. Furthermore, crosstalk between CAFs and ESCC cells can induce the expression and stimulation of FOXO1, which induces TGF-β1 production through the autocrine/paracrine feedback mechanism, leading to treatment resistance. TGF-β1 expression in CAFs is significantly linked to the OS of patients receiving chemoradiotherapy. Therefore, TGF-β1 in CAFs is a desirable target for reversing chemoresistance. Hence, it can be utilized as a standalone prognostic factor for patients with ESCC undergoing chemoradiotherapy. A frequently occurring cytokine called IL-6 promotes communication between tumour cells and their host microenvironment. Qiao et al. (66) reported that IL-6 released by CAFs was associated with ESCC cells’ resistance to cisplatin. IL-6 upregulates CXCR7 expression via the STAT3/NF-κB pathway, promoting ESCC cell proliferation and drug resistance. In addition to directly activating the ESCC cells’ drug resistance phenotype, IL-6 produced by CAFs also indirectly encourages drug resistance by triggering immunosuppressive M-MDSCs. Zhao et al. (44) demonstrated that IL-6 released by CAFs and RNA-21 activated activator of transcription 3 to promote the production of M-MDSCs. M-MDSCs induced by CAFs promoted cisplatin resistance in tumour cells. From CAFs pre-treated with cisplatin, Che et al.’s study (67) identified PAI-1, which is a key cytokine. PAI-1 in the TME promotes tumour progression and attenuates cisplatin’s therapeutic effects. Caspase-3 activity and the buildup of reactive oxygen species are inhibited by paracrine PAI-1 activating the AKT and ERK1/2 signalling pathways. Patients with ESCC with high PAI-1 expression in CAFs have significantly poor Progression-Free-Survival (PFS). As was already noted, exogenous lncRNA POU3F3, which is produced by ESCC cells and regulates fibroblast activation, can go from ESCC cells to NFs through exosomes. By secreting IL-6, activated fibroblasts aid in ESCC cells’ progression and cisplatin resistance.

CAFs secrete multiple cytokines and chemokines that interact with tumour cells to promote tumour metastasis, progression, and treatment resistance. CAFs are crucial in regulating how ESCC cells react to chemotherapy. Therefore, it is the high time to explore the molecular mechanism of their tumour-promoting activity.

Radiotherapy as a neoadjuvant and radical treatment, which is also significant. The 5-year survival rate of patients following radiation is less than 20% due to radiotherapy resistance. Increasing the radiosensitivity of tumours is beneficial in further improving the efficacy of treatment,consequently achieving better control of the tumour,leading to prolong the OS of patients with ESCC. As radiogenic cells, CAFs enter senescence and release various cytokines and chemokines that facilitate tumour cells survive after radiation. Furthermore, CAFs can trigger EMT, ECM remodelling and autophagy in tumour cells, which makes them resistant to radiation.

Chemokines and cytokines is significant in the interaction between CAFs and tumour cells. Zhang et al. (68) used a human chemokine/cytokine array and observed high expression levels of CXCL1 in CAFs. CXCL1 secreted by CAFs inhibited the expression of superoxide dismutase-1, resulting in raised ROS accumulation after radiation, enhanced DNA damage repair and mediated radiation resistance. In addition, CXCL1 secreted by CAFs mediated radiation resistance by activating the MEK/ERK pathway. The crosstalk between CAFs and ESCC cells further enhances radiation resistance by inducing CXCL1 expression via an autocrine/paracrine signalling pathway.

The expression of lncRNAs are aberrant in multiple human diseases, such as cancer (69). lncRNAs participate in ESCC (42) by detecting DNA damage, repairing damaged DNA, transmitting damage signals, triggering the cell cycle checkpoints and causing cell apoptosis (70). By modulating DDR, Zhang et al. (71) found that lncRNA DNM3OS significantly induced radiation resistance *in vitro* and vivo. CAFs enhanced the expression of DNM3OS through the PDGFb/PDGFRb/FOXO-1 signalling pathway.

**Figure 2:**
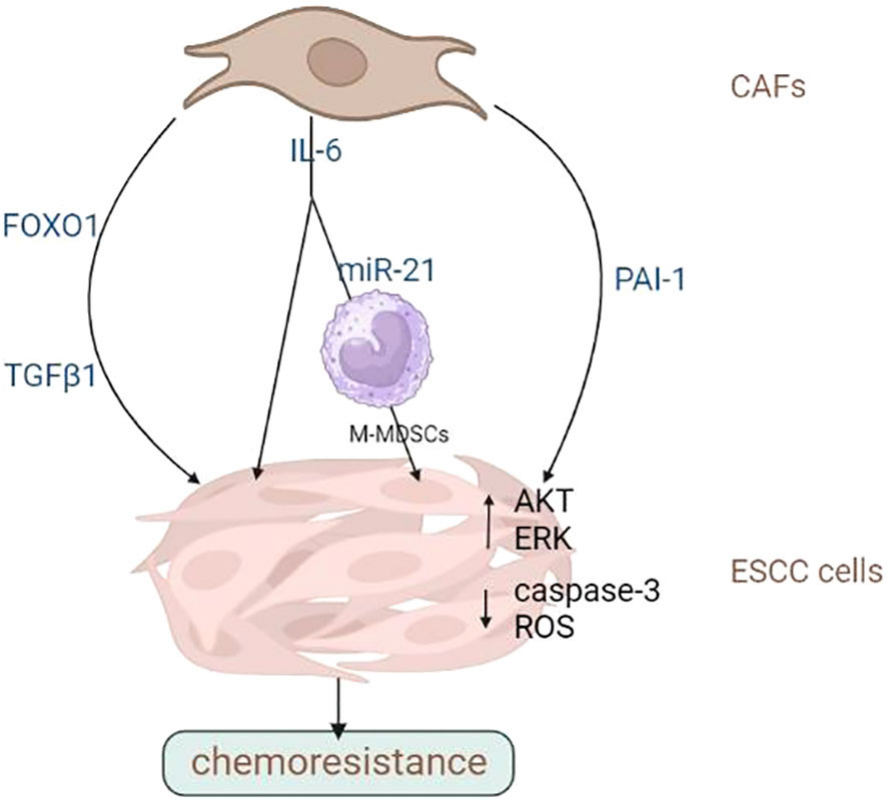
CAFs promote the chemoresistance of ESCC cells. CAFs contribute to drug resistance and tumour metastasis through multiple factors. Image created with BioRender.com.

**Figure 3:**
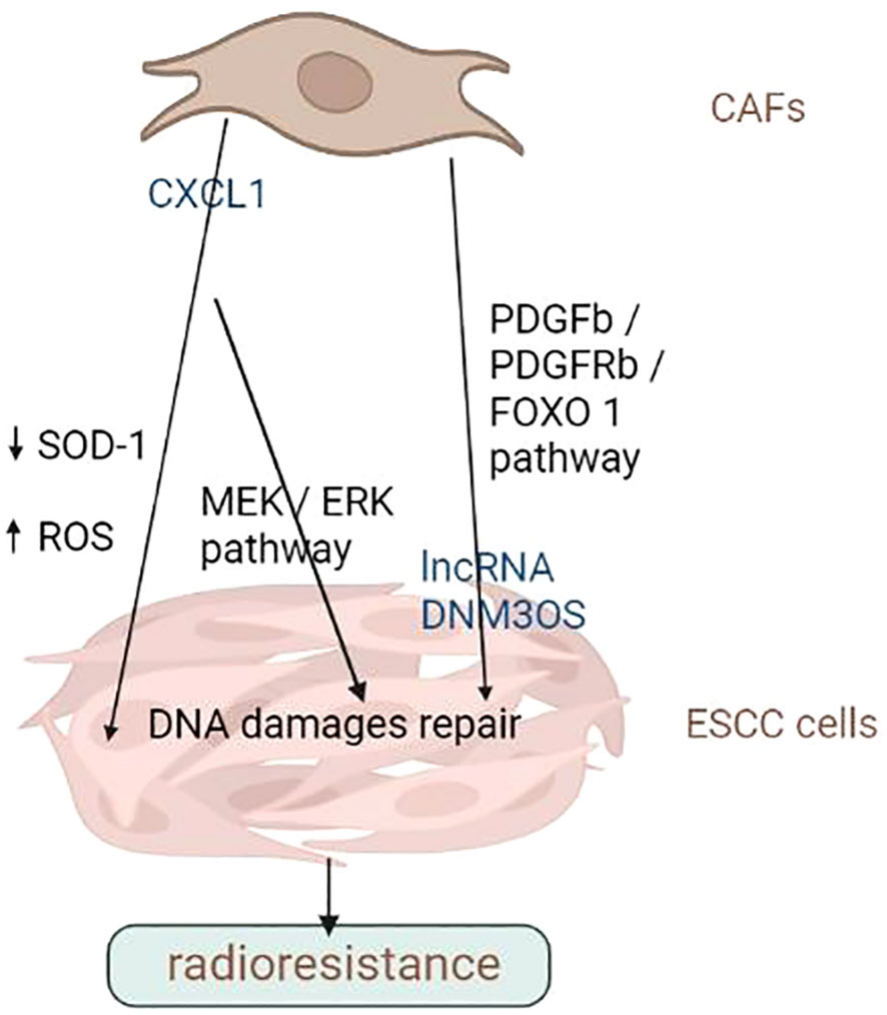
CAFs promote the radiation resistance of ESCC cells. CAFs can secrete cytokines, chemokine and growth factors that promote tumour cell survival and proliferation or activate signalling pathways that lead to radiation resistance. Image created with BioRender.com.

Altogether, CAFs is critical in determining the outcome of ESCC cells to chemotherapy and radiation therapy. They can secrete cytokines, chemokine and growth factors that promote tumour cell survival and proliferation or activate signalling pathways that lead to resistance to chemotherapy or radiotherapy. CAFs can remodel the ECM, impairing immune cell infiltration or making it more difficult for drugs to penetrate the tumour. In addition, CAFs can stimulate tumour angiogenesis or induce EMT, leading to higher aggressiveness and metastasis. Therefore, targeting CAFs is a potential approach for overcoming treatment resistance in various cancer. In-depth studies are warranted to investigate the molecular basis of CAFs’ tumour-promoting activities and develop novel treatment strategies targeting them to overcome drug resistance in patients with advanced ESCC. Overall, CAFs are potential targets for reversing chemoresistance and improving OS rates in ESCC. The promotion of chemo- and radio-resistance of ESCC cells by CAFs was summarized in **Figures 2**, **3** and **Table 2**.

**Table 2:** Role of CAFs in treatment resistance in ESCC.

Treatment resistance	Mediators	Results	References
Chemoresistance	TGF-β1	induces the expression and activation of FOXO1, which induces TGF-β1 expression through an autocrine/paracrine feedback loop, leading to treatment resistance	Zhang et al. (65)
	IL-6	upregulates CXCR7 expression through the STAT3/NF-κB pathway, promoting ESCC cell proliferation and drug resistance	Qiao et al. (66)
		activates activator of transcription 3 to promote the production of M-MDSCs, promoting resistance to cisplatin in tumour cells	Zhao et al. (44)
	PAI-1	promotes tumour growth and attenuates the therapeutic effects of cisplatin	Che et al. (67)
	exogenous lncRNA POU3F3	promotes the proliferation and cisplatin resistance of ESCC cells by secreting IL-6	Tong et al. (27)
Radiotherapy resistance	CXCL1	increases ROS accumulation after radiation, enhances DNA damage repair and mediates radiation resistance by activating the MEK/ERK pathway	Zhang et al. (68)
	lncRNA DNM3OS	conferres significant radiation resistance *in vitro* and *in vivo* by regulating DDR and the PDGFb/PDGFRb/FOXO-1 signalling pathway	Zhang et al. (71)

## 5 Value of CAFs in predicting ESCC prognosis

Identifying prognostic markers for EC is important because the disease is life-threatening and has an impact on treatment efficacy(**Table 3**). Proteins identified in CAFs may function as prognostic indicators for EC in addition to their roles in carcinogenesis, proliferation, angiogenesis,migration, invasion, and dissemination. Immature CAF phenotypes and alpha smooth muscle actin (α-SMA), a CAF marker, have been concerned in the lower survival of patients with ESCC and EAC, respectively (60, 80).

**Table 3:** CAFs are potential pathological indicators of EC prognosis.

Histological subtypes	Selection of cases	Results			Prognosis	Predictive value	Reference
ESCC	95 patients with ESCC who underwent oesophagectomy (2007)		3-year OS (%)		Inverse correlation with OS	CAF density may serve as a marker for predicting therapeutic efficacy and prognosis	Cheng et al. (72)
		CAF-poor group	63				
		CAF-rich group	42				
	153 ESCC samples	Increased CD10 expression was associated with poor tumour differentiation, OS and DFS				CD10 overexpression may serve as an independent poor prognostic factor	Lee et al. (73)
	51 tumour sample from ESCC patients who underwent surgery without preoperative treatment		Median survival time (months)		CAF-derived Wnt2 was significantly associated with lymph node metastasis, and patients with high Wnt2 expression had shorter median survival time	Wnt2 promotes tumour growth and invasion	Fu et al. (74)
		Wnt2-positive ESCC	16				
		Wnt2-negative ESCC	51				
	102 ESCC samples	Patients in the high-TGFBI-expression group (n = 16) had more frequent haematogenous recurrence than the low-TGFB1-expression group (n = 86)				TGFβI expression may serve as an independent predictor of OS.	Ozawa et al. (75)
		FAP+ CAFs were strongly associated with lymph node metastasis				CAFs may serve as a prognostic factor	Kashima et al. (76)
	A total of 70 surgically removed human ESCC tissue samples were collected between 2005 and 2010	The expression of two CAF markers, α-SMA and FAP, was linked to the depth of tumour invasion, lymph node metastasis, advanced clinical stage, progressed pathological stage and poor prognosis					Higashino et al. (42)
	152 patients	Overexpression of LTBP1 was positively linked to lymphatic metastasis in ESCC (p = 0.002)				LTBP1 plays an oncogenic role in ESCC progression and may serve as a potential therapeutic target for ESCC	Cai et al. (77)
	Patients with ESCC who underwent surgical resection	HIC-5 overexpression in the tumour stroma was associated with positive lymph node metastases and a higher TNM stage				HIC-5 is a risk factor for lymph node metastasis in ESCC	Du et al. (30)
		High PAI-1 expression was associated with poor PFS				PAI-1 in CAFs is a potential prognostic factor for ESCC	(35, 67)
		Increased stromal uPA levels (132/146 cases) were associated with tumour invasion (p < 0.05) and OS (p < 0.05) in ESCC				uPA may serve as a predictive marker for the diagnosis and prognosis of ESCC and an effective therapeutic target	Tian et al. (34)
	189 formalin-fixed and paraffin-embedded tissue samples	A strong correlation was observed between poor clinical outcomes and the concurrent expression of Twist1 and other CAF markers				Twist1 may serve as a novel CAF marker for predicting the prognosis of ESCC and is a potent therapeutic target	Yeo et al. (78)
	171 patients	High POSTN expression was associated with poor OS (P = 0.0001) and RFS (P = 0.03).				High POSTN expression is an independent prognostic factor for ESCC	Ishibashi et al. (79)
EAC	183 patients with EAC			OS (months)	DFS (months)	POSTN may be an independent prognostic factor for EAC	Underwood et al. (80)
		POSTN-positive tumours		46.8	47.45		
		POSTN-negative tumours		76.45	80.67		
	200 patients with EAC who underwent surgery	Podoplanin-expressing CAFs were positively associated with short OS (42 versus 105 months) and mean DFS (42 versus 89 months)					Schoppmann et al. (81)

Cheng et al. (72) collected samples from 95 patients with ESCC who underwent oesophagectomy and stained them with an SMA-specific antibody to evaluate the abundance of CAFs.In comparison to the CAF-rich group, which had a 3-year OS rate of 42%(P<0.01), the CAF-poor group had a 3-year OS rate of 63% (P<0.01). According to univariate and multivariate Cox analyses of 3-year OS, the hazard ratio of CAF density in the CAF-poor group was 1.870 (95% CI, 1.033–3.385; P = 0.039), while CAF-rich group’ data was 2.196 (95% CI, 1.150–4.193; P = 0.017), indicating that CAF density was a reliable independent predictor of 3-year OS. These findings suggest that CAF density serves as a marker for predicting therapeutic efficacy and prognosis in ESCC. Increased CD10 expression in ESCC patients has been in correlation with inferior tumour differentiation, OS and disease-free survival (DFS) (73). Fu et al. (74) researched 51 tumour samples from ESCC patients who underwent surgery without receiving prior care. CAF-derived Wnt2 was significantly associated with lymph node metastasis. Patients with high Wnt expression also had a shorter median survival time (16 months) than patients with Wnt2-negative ESCC (16 months). According to Ozawa et al.’s research (75), the protein expression of TGFβI was enhanced in fibroblasts, and this elevated expression served as an independent predictor of OS. Lymph node metastasis is a characteristic characteristic of cancer, and combating metastasis to achieve better therapeutic outcomes is difficult. Lymph node metastasis is a significant poor prognostic factor for EC. Fibroblast-activating protein (FAP), a CAF marker, has been positively connected with lymph node metastasis in clinical samples (76). Higashino et al. (42) subjected ESCC tissues to immunohistochemical staining and observed a significant correlation between the production of two CAF markers, α-SMA and FAP, and the depth of tumour invasion, lymph node metastasis, advanced clinical stage, progressed pathological stage and poor prognosis. The chromosomal band 2p22.3 contains latent transforming growth factor β-binding protein 1 (LTBP1), which takes role in the formation and release of latent TGF-β1. In addition, LTBP1 contributes significantly to tumourigenesis. In ESCC, lymphatic metastasis has been favorably linked to overexpression of LTBP1 (77). In CAFs isolated from the tumor stroma of ESCC patients, HIC-5 is substantially expressed. Hign HIC-5 expression in the tumour stroma has been linked to tumour invasion, lymph node metastases and advanced pathological stage. Du et al. (30) recognized stromal HIC-5 as a risk factor for lymph node metastasis in ESCC. Treatment strategies targeting CAFs can lessen lymph node metastasis and enhance EC patients’ prognosis. Patients with ESCC who express high PAI-1 in CAFs have significantly poorer PFS (67). Additionally, a poor prognosis in ESCC has been linked to elevated PAI-1 and LRP1 expression (35). Tian et al. (34) used an antibody array and identified uPA whose release was higher in CAFs than in NFs. Increased stromal uPA levels (132/146 cases) were associated with tumour invasion (p < 0.05) and OS (p < 0.05) in patients with ESCC. Yeo et al. (78) demonstrated a favourable association between Twist1 expression and CAF markers including PDGFR, α-SMA, tenascin-C and FSP1. Additionally, there was a substantial correlation between poor clinical outcomes with the simultaneous expression of Twist1 and other CAF markers. POSTN, which actively contributes to inflammation and carcinogenesis, is largely released by CAFs in the TME. A study (79) reported a direact correlation between high POSTN expression and poor OS (P = 0.0001) and recurrence-free survival (RFS) (P = 0.03).

The abovementioned studies suggest that CAFs and some factors secreted by CAFs can serve as potential prognostic markers for ESCC.

In a study, CAF-derived POSTN was identified as a reliable indicator of survival in EAC patients. An increase in α-SMA expression resulted in an increase in POSTN expression, suggesting that CAFs released POSTN. In addition, OS (46.80 versus 76.45 months) and DFS (47.45 versus 80.67 months) were shorter among patients with POSTN-positive tumours than among those with POSTN-negative tumours (80). The transmembrane sialoglycoprotein podoplanin may serve as a prognostic factor for EAC. Schoppmann et al. (81) reported that podoplanin-expressing CAFs had a positive connection with shorter OS (42 versus 105 months) and mean DFS (42 versus 89 months) in patients with EAC who underwent surgery. Altogether, the high expression of the abovementioned proteins predicted a adverse prognosis irrespective of the disease.

## 6 Application of CAFs in the treatment of ESCC

Owing to their multiple tumour-promoting functions, CAFs represent promising therapeutic targets for cancer. Theoretically, either directly targeting CAFs to deplete and eliminate them or reprogramming CAFs to a normal fibroblast phenotype can inhibit their tumour-promoting effects. In addition, indirectly inhibiting the communication between CAFs and neighbouring cells attenuates the tumour-promoting fuctions of CAFs. Although several promising results have been reported, CAF-targeting therapy is challenging owing to the heterogeneity of CAFs and the lack of CAF-specific markers.

### 6.1 Direct depletion or inactivation of CAFs

Numerous studies have attempted to develop strategies for inactivating or depleting CAFs. These strategies mostly target indicators present on the CAF surface, such as FAP and α-SMA. ESCC has been successfully treated with targeted therapeutic strategies that incorporate FAP, a typical CAF biomarker (82). Various therapeutic formulations, some of which are undergoing clinical trials, have been developed to target FAP-positive CAFs, including DNA vaccines, tumour-lysing adenoviruses and nanoparticles (83–85). Regarding nanoparticles, Zhen et al. used ferritin to bind to particular single chain variable segments of FAP. Nanoparticle-based photoimmunotherapy(nano-PIT), is the name of this method. Nano-PIT directly and successfully destroys cancer cells while sparing healthy tissue from harm. Additionally, nanoparticles reduce CXCL12 production and ECM deposition to control t-cell rejection, which effectively suppresses tumor growth. The utilization of direct targeting of nanoparticles for CAF treatment is promising and safe. Hence, eliminating the tumour-promoting effects by targeting FAP -positive CAFs is a viable strategy that may improve the prognosis of patients with ESCC.

Katsube et al. (86) devised FAP-targeted near-infrared photoimmunotherapy (NIR-PIT). NIR-PIT is an innovative method that can be utilized to precisely and directly deplete FAP-postive CAFs in the TME. NIR-PIT prevented tumour growth *in vivo* without resulting in negative consequences (87). Compared with 5-fluorouracil (5-FU) monotherapy, combination therapy with anti-FAP + CAFs and 5-FU can overcome chemoresistance. In addition, dual-targeted NIR-PIT has been used in other studies (88) for attacking both tumour cells and CAFs. It has demonstrated better therapeutic effects *in vitro* and vivo compared with single-targeted NIR-PIT. Therefore, dual-targeted NIR-PIT might represent an effective cancer treatment technique.

The high expression of FAP in CAFs can aid in the advancement of pan-tumour molecular visualization methods in contrast to having therapeutic implications (89). In recent studies, the use of gallium-68-labelled small-molecule FAP inhibitors (FAPIs) as a PET tracer has demonstrated advantages over fluorodeoxyglucose PET in the detection of malignancy in patients containing FAP+ cells (90, 91). Furthermore, FAPI-PET offers a better representation of the objective volume for scheduling radiation therapy (92). In light of this, FAPI-PET, a unique molecular visualization tool that can be utilized to predict the therapeutic outcome of anti-FAP CAF-targeting therapy, may improve the diagnosis and treatment of ESCC.

A disadvantage limiting the accuracy of the abovementioned strategies is that none of the surface markers, such as FAP, are uniquely expressed by fibroblasts. In this context, fibroblast-specific surface proteins such as CD10 and GPR77 can serve as unique human CAF subpopulations with pro-tumour functions (93). Therefore, identifying more accurate markers is necessary for more effective targeting and better protection of normal cells.

Preclinical and clinical studies have demonstrated that some natural products can directly or indirectly target CAFs to exert therapeutic effects. These products interfere with the activation, differentiation and tumour-promoting functions of CAFs and tumour–stromal crosstalk. In addition, they can inhibit ECM remodelling and the paracrine function of CAFs (94). However, most studies investigating the potential of natural products in targeting CAFs are at the experimental stage. Several technical challenges, including as bioavailability, water solubility, and accuracy, limit the availability of these products.

CAFs play an integral function in promoting tumour progression. Nevertheless, clinical trials verifying the effectiveness of directly targeting CAFs are lacking, the genesis and functional significance of unique CAF subpopulations remain unclear and their ecological niches differ in various tumour types. Therefore, for rapid development of CAF-specific diagnostic or prognostic protocols and CAF-targeting therapies, in-depth genome sequencing should be used to understand the molecular landscape of fibroblasts. Although most biological characteristics of CAFs have been modelled *in vitro*, it has been consistently established that CAFs in culture do not completely mimic CAF heterogeneity *in vivo* (95–97). Over time, the addition of animal models may provide further insights into the origin, formation, plasticity and phenotype of CAFs. To this end, emerging technologies like single-cell RNA sequencing (98–101) should be used to identify additional subpopulations of CAFs and their cellular interactions and understand the heterogeneity and plasticity of CAFs. In addition, these techniques can be used to selectively eliminate subpopulations of pro-tumour CAFs or reverse their pro-tumour activity. These strategies may be used alone or in conjuction with other strategies of therapy. The development of novel technologies may facilitate the identification of more precise targeted therapies.

### 6.2 Neutralisation of functional molecules secreted by CAFs

The neutralization of functional components secreted by CAFs is a different approach for eradicating their tumour-promoting activities, as opposed to direct diminution or elimination of CAFs. Many studies have identified functional molecules of CAFs as potential targets for inhibiting the progression of EC.

IL-6, an important cytokine released by CAFs, can promote the development of ESCC. It mediates the interaction between tumour cells and CAFs by boosting tumour cell progression as well as by promoting fibroblast activation. Karakasheva et al. (26) found that loss of IL-6 inhibited tumourigenesis *in vitro* and *in vivo*, suggesting that IL-6 receptors and downstream effectors represent promising targets for the treatment of upper gastrointestinal cancers. Novel inhibitors targeting IL-6, the IL-6 receptor or signalling molecules related to IL-6 have entered clinical and/or preclinical trials involving patients with cancer (102–105).

Additionally, SDF-1 frequently acts as a mediator in the interaction between CAFs and tumour cells. Clinical investigations have assessed the antitumour effects of inhibiting CAF-secreted SDF-1 signaling (106, 107).

Blocking CAF- released molecules, like IL-6, can eliminate the pro-tumourigenic effects of CAFs and help to overcome drug resistance and improve prognosis in patients treated with chemotherapy or radiotherapy. Overall, drugs that target CAF signalling and effectors have become a significant addition to cancer-specific therapies for a broad variety of solid tumours. The therapeutic efficacy of numerous agents in cancers such as lung cancer, rectal cancer, head and neck cancer and pancreatic ductal carcinoma is being researched in preclinical and clinical trials. However, clinical trials involving patients with EC are less frequently performed, and further clinical investigations are required to optimize the theoretical basis of treatment. In addition, more specific druggable molecular targets that modulate CAF signalling should be examined in mechanistic and functional studies.

Altogether, targeting CAFs is a promising approach to treating EC. CAFs is significant in promoting tumour growth and have been shown to contribute to treatment resistance. Direct depletion or inactivation of CAFs by targeting surface markers like FAP represents a potential therapeutic strategy. In addition, natural compounds have shown promising outcomes in preclinical and clinical studies. The tumour-promoting effects of CAFs can be reduced by indirectly neutralising some functional molecules of CAFs. However, the heterogeneity of CAFs remains a major obstacle to achieving precise targeting. Novel technologies including single-cell sequencing and bioinformatics can assist researchers better understand of heterogeneity of CAFs and facilitate the development of more precise targeted therapies. Overall, developing therapeutic strategies targeting CAFs is important for improving treatment outcomes in EC.

## 7 Conclusion

CAFs play an important role in the TME. The phenotype, characteristics and heterogeneity of CAFs have been investigated in many studies. This review provided insights into the origin and activation of CAFs and summarised the various roles of CAFs in modulating tumourigenesis and treatment resistance. Numerous CAF-targeting therapies are undergoing clinical or preclinical trials at present.

However, the heterogeneity of CAFs is a major challenge to treatment, and advanced technologies such as single-cell sequencing and bioinformatics should be used to improve our understanding of CAFs. These techniques can help identify specific subtypes of CAFs with different functions, thereby contributing to a better understanding of the interactions between CAFs and other components in the TME. Future studies are supposed to focus on elucidating the exact mechanisms underlying treatment resistance and the interaction between specific CAF subtypes and different molecules. An in-depth understanding of CAFs may aid to design or refine risk and prognostic models of EC. Overall, continuous investigation of CAFs is necessary for advancing the existing knowledge on tumour biology and improving treatment outcomes.

## Author contributions

MX: Writing – original draft. YT: Writing – review & editing. YX: Writing – review & editing. CY: Writing – review & editing.

## References

[1] Sung H Ferlay J Siegel RL Laversanne M Soerjomataram I Jemal A. Global cancer statistics 2020: GLOBOCAN estimates of incidence and mortality worldwide for 36 cancers in 185 countries. CA Cancer J Clin (2021) 71(3):209–49. doi: 10.3322/caac.21660 33538338

[2] Murphy G McCormack V Abedi-Ardekani B Arnold M Camargo MC Dar NA. International cancer seminars: a focus on esophageal squamous cell carcinoma. Ann Oncol (2017) 28(9):2086–93. doi: 10.1093/annonc/mdx279 PMC5834011 28911061

[3] Herskovic A Russell W Liptay M Fidler MJ Al-Sarraf M. Esophageal carcinoma advances in treatment results for locally advanced disease: review. Ann Oncol (2012) 23(5):1095–103. doi: 10.1093/annonc/mdr433 22003242

[4] di Pietro M Canto MI Fitzgerald RC. Endoscopic management of early adenocarcinoma and squamous cell carcinoma of the esophagus: screening, diagnosis, and therapy. Gastroenterology (2018) 154(2):421–36. doi: 10.1053/j.gastro.2017.07.041 PMC6104810 28778650

[5] Waters JK Reznik SI. Update on management of squamous cell esophageal cancer. Curr Oncol Rep (2022) 24(3):375–85. doi: 10.1007/s11912-021-01153-4 35142974

[6] Montagnani F Fornaro L Frumento P Vivaldi C Falcone A Fioretto L. Multimodality treatment of locally advanced squamous cell carcinoma of the oesophagus: a comprehensive review and network meta-analysis. Crit Rev Oncol Hematol (2017) 114:24–32. doi: 10.1016/j.critrevonc.2017.03.024 28477744

[7] Leng XF Daiko H Han YT Mao YS. Optimal preoperative neoadjuvant therapy for resectable locally advanced esophageal squamous cell carcinoma. Ann N Y Acad Sci (2020) 1482(1):213–24. doi: 10.1111/nyas.14508 33067818

[8] Pennathur A Gibson MK Jobe BA Luketich JD. Oesophageal carcinoma. Lancet (2013) 381(9864):400–12. doi: 10.1016/S0140-6736(12)60643-6 23374478

[9] Turley SJ Cremasco V Astarita JL. Immunological hallmarks of stromal cells in the tumour microenvironment. Nat Rev Immunol (2015) 15(11):669–82. doi: 10.1038/nri3902 26471778

[10] Bruni D Angell HK Galon J. The immune contexture and Immunoscore in cancer prognosis and therapeutic efficacy. Nat Rev Cancer (2020) 20(11):662–80. doi: 10.1038/s41568-020-0285-7 32753728

[11] Zhou J Kryczek I Li S Li X Aguilar A Wei S. The ubiquitin ligase MDM2 sustains STAT5 stability to control T cell-mediated antitumor immunity. Nat Immunol (2021) 22(4):460–70. doi: 10.1038/s41590-021-00888-3 PMC8026726 33767425

[12] Zou W. Immune regulation in the tumor microenvironment and its relevance in cancer therapy. Cell Mol Immunol (2022) 19(1):1–2. doi: 10.1038/s41423-021-00738-0 34992273 PMC8752764

[13] Kalluri R. The biology and function of fibroblasts in cancer. Nat Rev Cancer (2016) 16(9):582–98. doi: 10.1038/nrc.2016.73 27550820

[14] Polyak K Haviv I Campbell IG. Co-evolution of tumor cells and their microenvironment. Trends Genet (2009) 25(1):30–8. doi: 10.1016/j.tig.2008.10.012 19054589

[15] Biffi G Tuveson DA. Diversity and biology of cancer-associated fibroblasts. Physiol Rev (2021) 101(1):147–76. doi: 10.1152/physrev.00048.2019 PMC7864232 32466724

[16] Mao X Xu J Wang W Liang C Hua J Liu J. Crosstalk between cancer-associated fibroblasts and immune cells in the tumor microenvironment: new findings and future perspectives. Mol Cancer (2021) 20(1):131. doi: 10.1186/s12943-021-01428-1 34635121 PMC8504100

[17] Ishii G Ochiai A Neri S. Phenotypic and functional heterogeneity of cancer-associated fibroblast within the tumor microenvironment. Adv Drug Delivery Rev (2016) 99(Pt B):186–96. doi: 10.1016/j.addr.2015.07.007 26278673

[18] Mezawa Y Orimo A. Phenotypic heterogeneity, stability and plasticity in tumor-promoting carcinoma-associated fibroblasts. FEBS J (2022) 289(9):2429–47. doi: 10.1111/febs.15851 33786982

[19] Mishra PJ Mishra PJ Humeniuk R Medina DJ Alexe G Mesirov JP. Carcinoma-associated fibroblast-like differentiation of human mesenchymal stem cells. Cancer Res (2008) 68(11):4331–9. doi: 10.1158/0008-5472.CAN-08-0943 PMC2725025 18519693

[20] Huang L Xu AM Liu S Liu W Li TJ. Cancer-associated fibroblasts in digestive tumors. World J Gastroenterol (2014) 20(47):17804–18. doi: 10.3748/wjg.v20.i47.17804 PMC4273131 25548479

[21] Ganguly D Chandra R Karalis J Teke M Aguilera T Maddipati R. Cancer-associated fibroblasts: versatile players in the tumor microenvironment. Cancers (Basel) (2020) 12(9):2652. doi: 10.3390/cancers12092652 32957515 PMC7564346

[22] Roy A Bera S. CAF cellular glycolysis: linking cancer cells with the microenvironment. Tumour Biol (2016) 37(7):8503–14. doi: 10.1007/s13277-016-5049-3 27075473

[23] Chen X Song E. Turning foes to friends: targeting cancer-associated fibroblasts. Nat Rev Drug Discovery (2019) 18(2):99–115. doi: 10.1038/s41573-018-0004-1 30470818

[24] Fang L Che Y Zhang C Huang J Lei Y Lu Z. LAMC1 upregulation *via* TGFbeta induces inflammatory cancer-associated fibroblasts in esophageal squamous cell carcinoma *via* NF-kappaB-CXCL1-STAT3. Mol Oncol (2021) 15(11):3125–46. doi: 10.1002/1878-0261.13053 PMC8564640 34218518

[25] Chen Y Zhu S Liu T Zhang S Lu J Fan W. Epithelial cells activate fibroblasts to promote esophageal cancer development. Cancer Cell (2023) 41(5):903–918.e8. doi: 10.1016/j.ccell.2023.03.001 36963399

[26] Karakasheva TA Lin EW Tang Q Qiao E Waldron TJ Soni M. IL-6 mediates cross-talk between tumor cells and activated fibroblasts in the tumor microenvironment. Cancer Res (2018) 78(17):4957–70. doi: 10.1158/0008-5472.CAN-17-2268 PMC6125177 29976575

[27] Tong Y Yang L Yu C Zhu W Zhou X Xiong Y. Tumor-Secreted Exosomal lncRNA POU3F3 Promotes Cisplatin Resistance in ESCC by Inducing Fibroblast Differentiation into CAFs. Mol Ther Oncolytics (2020) 18:1–13. doi: 10.1016/j.omto.2020.05.014 32637576 PMC7321817

[28] Fang L Che Y Zhang C Huang J Lei Y Lu Z. PLAU directs conversion of fibroblasts to inflammatory cancer-associated fibroblasts, promoting esophageal squamous cell carcinoma progression *via* uPAR/Akt/NF-kappaB/IL8 pathway. Cell Death Discovery (2021) 7(1):32. doi: 10.1038/s41420-021-00410-6 33574243 PMC7878926

[29] Chen J Wang Y Zhang W Zhao D Zhang L Zhang J. NOX5 mediates the crosstalk between tumor cells and cancer-associated fibroblasts *via* regulating cytokine network. Clin Transl Med (2021) 11(8):e472. doi: 10.1002/ctm2.472 34459125 PMC8329696

[30] Du X Xu Q Pan D Xu D Niu B Hong W. HIC-5 in cancer-associated fibroblasts contributes to esophageal squamous cell carcinoma progression. Cell Death Dis (2019) 10(12):873. doi: 10.1038/s41419-019-2114-z 31740661 PMC6861248

[31] Shimizu M Koma YI Sakamoto H Tsukamoto S Kitamura Y Urakami S. Metallothionein 2A expression in cancer-associated fibroblasts and cancer cells promotes esophageal squamous cell carcinoma progression. Cancers (Basel) (2021) 13(18):4552. doi: 10.3390/cancers13184552 34572779 PMC8464741

[32] Ishibashi Y Mochizuki S Horiuchi K Tsujimoto H Kouzu K Kishi Y. Periostin derived from cancer-associated fibroblasts promotes esophageal squamous cell carcinoma progression *via* ADAM17 activation. Biochim Biophys Acta Mol Basis Dis (2023) 1869(5):166669. doi: 10.1016/j.bbadis.2023.166669 36813090

[33] Dunbar KJ Karakasheva TA Tang Q Efe G Lin EW Harris M. Tumor-derived CCL5 recruits cancer-associated fibroblasts and promotes tumor cell proliferation in esophageal squamous cell carcinoma. Mol Cancer Res (2023) 21(7):741–52. doi: 10.1158/1541-7786.MCR-22-0872 PMC10330279 37027010

[34] Tian B Chen X Zhang H Li X Wang J Han W. Urokinase plasminogen activator secreted by cancer-associated fibroblasts induces tumor progression *via* PI3K/AKT and ERK signaling in esophageal squamous cell carcinoma. Oncotarget (2017) 8(26):42300–13. doi: 10.18632/oncotarget.15857 PMC5522068 28404945

[35] Sakamoto H Koma YI Higashino N Kodama T Tanigawa K Shimizu M. PAI-1 derived from cancer-associated fibroblasts in esophageal squamous cell carcinoma promotes the invasion of cancer cells and the migration of macrophages. Lab Invest (2021) 101(3):353–68. doi: 10.1038/s41374-020-00512-2 PMC7892342 33311557

[36] Jin Y Meng Q Zhang B Xie C Chen X Tian B. Cancer-associated fibroblasts-derived exosomal miR-3656 promotes the development and progression of esophageal squamous cell carcinoma *via* the ACAP2/PI3K-AKT signaling pathway. Int J Biol Sci (2021) 17(14):3689–701. doi: 10.7150/ijbs.62571 PMC8495391 34671193

[37] Shi Z Jiang T Cao B Sun X Liu J. CAF-derived exosomes deliver LINC01410 to promote epithelial-mesenchymal transition of esophageal squamous cell carcinoma. Exp Cell Res (2022) 412(2):113033. doi: 10.1016/j.yexcr.2022.113033 35041823

[38] Chen C Yang C Tian X Liang Y Wang S Wang X. Downregulation of miR-100-5p in cancer-associated fibroblast-derived exosomes facilitates lymphangiogenesis in esophageal squamous cell carcinoma. Cancer Med (2023) 12(13):14468–83. doi: 10.1002/cam4.6078 PMC10358253 37184125

[39] Wang Z Zhang M Liu L Yang Y Qiu J Yu Y. Prognostic and immunological role of cancer-associated fibroblasts-derived exosomal protein in esophageal squamous cell carcinoma. Int Immunopharmacol (2023) 124(Pt A):110837. doi: 10.1016/j.intimp.2023.110837 37634448

[40] Zhao G Li H Guo Q Zhou A Wang X Li P. Exosomal Sonic Hedgehog derived from cancer-associated fibroblasts promotes proliferation and migration of esophageal squamous cell carcinoma. Cancer Med (2020) 9(7):2500–13. doi: 10.1002/cam4.2873 PMC7131837 32030915

[41] Qiu L Yue J Ding L Yin Z Zhang K Zhang H. Cancer-associated fibroblasts: An emerging target against esophageal squamous cell carcinoma. Cancer Lett (2022) 546:215860. doi: 10.1016/j.canlet.2022.215860 35948121

[42] Higashino N Koma YI Hosono M Takase N Okamoto M Kodaira H. Fibroblast activation protein-positive fibroblasts promote tumor progression through secretion of CCL2 and interleukin-6 in esophageal squamous cell carcinoma. Lab Invest (2019) 99(6):777–92. doi: 10.1038/s41374-018-0185-6 30683902

[43] Cui G Li C Xu G Sun Z Zhu L Li Z. Tumor-associated fibroblasts and microvessels contribute to the expression of immunosuppressive factor indoleamine 2, 3-dioxygenase in human esophageal cancers. Pathol Oncol Res (2018) 24(2):269–75. doi: 10.1007/s12253-017-0244-0 28470572

[44] Zhao Q Huang L Qin G Qiao Y Ren F Shen C. Cancer-associated fibroblasts induce monocytic myeloid-derived suppressor cell generation *via* IL-6/exosomal miR-21-activated STAT3 signaling to promote cisplatin resistance in esophageal squamous cell carcinoma. Cancer Lett (2021) 518:35–48. doi: 10.1016/j.canlet.2021.06.009 34139285

[45] Kato T Noma K Ohara T Kashima H Katsura Y Sato H. Cancer-associated fibroblasts affect intratumoral CD8(+) and foxP3(+) T cells *via* IL6 in the tumor microenvironment. Clin Cancer Res (2018) 24(19):4820–33. doi: 10.1158/1078-0432.CCR-18-0205 29921731

[46] Huang TX Tan XY Huang HS Li YT Liu BL Liu KS. Targeting cancer-associated fibroblast-secreted WNT2 restores dendritic cell-mediated antitumour immunity. Gut (2022) 71(2):333–44. doi: 10.1136/gutjnl-2020-322924 PMC8762012 33692094

[47] Chen QY Li YN Wang XY Zhang X Hu Y Li L. Tumor fibroblast-derived FGF2 regulates expression of SPRY1 in esophageal tumor-infiltrating T cells and plays a role in T-cell exhaustion. Cancer Res (2020) 80(24):5583–96. doi: 10.1158/0008-5472.CAN-20-1542 33093168

[48] Curtis M Kenny HA Ashcroft B Mukherjee A Johnson A Zhang Y. Fibroblasts mobilize tumor cell glycogen to promote proliferation and metastasis. Cell Metab (2019) 29(1):141–155.e9. doi: 10.1016/j.cmet.2018.08.007 30174305 PMC6326875

[49] Bauer DE Harris MH Plas DR Lum JJ Hammerman PS Rathmell JC. Cytokine stimulation of aerobic glycolysis in hematopoietic cells exceeds proliferative demand. FASEB J (2004) 18(11):1303–5. doi: 10.1096/fj.03-1001fje PMC4458073 15180958

[50] Kelly B O'Neill LA. Metabolic reprogramming in macrophages and dendritic cells in innate immunity. Cell Res (2015) 25(7):771–84. doi: 10.1038/cr.2015.68 PMC4493277 26045163

[51] Wang FT Sun W Zhang JT Fan YZ. Cancer-associated fibroblast regulation of tumor neo-angiogenesis as a therapeutic target in cancer. Oncol Lett (2019) 17(3):3055–65. doi: 10.3892/ol.2019.9973 PMC6396119 30867734

[52] Papoutsoglou P Louis C Coulouarn C. Transforming growth factor-beta (TGFbeta) signaling pathway in cholangiocarcinoma. Cells (2019) 8(9):960. doi: 10.3390/cells8090960 31450767 PMC6770250

[53] Mekkawy AH Pourgholami MH Morris DL. Involvement of urokinase-type plasminogen activator system in cancer: an overview. Med Res Rev (2014) 34(5):918–56. doi: 10.1002/med.21308 24549574

[54] Li Y Lu Z Che Y Wang J Sun S Huang J. Immune signature profiling identified predictive and prognostic factors for esophageal squamous cell carcinoma. Oncoimmunology (2017) 6(11):e1356147. doi: 10.1080/2162402X.2017.1356147 29147607 PMC5674952

[55] Kahlert C Kalluri R. Exosomes in tumor microenvironment influence cancer progression and metastasis. J Mol Med (Berl) (2013) 91(4):431–7. doi: 10.1007/s00109-013-1020-6 PMC4073669 23519402

[56] Zhang L Yu D. Exosomes in cancer development, metastasis, and immunity. Biochim Biophys Acta Rev Cancer (2019) 1871(2):455–68. doi: 10.1016/j.bbcan.2019.04.004 PMC6542596 31047959

[57] Davern M Donlon NE Power R Hayes C King R Dunne MR. The tumour immune microenvironment in oesophageal cancer. Br J Cancer (2021) 125(4):479–94. doi: 10.1038/s41416-021-01331-y PMC8368180 33903730

[58] Kojima Y Acar A Eaton EN Mellody KT Scheel C Ben-Porath I. Autocrine TGF-beta and stromal cell-derived factor-1 (SDF-1) signaling drives the evolution of tumor-promoting mammary stromal myofibroblasts. Proc Natl Acad Sci U.S.A. (2010) 107(46):20009–14. doi: 10.1073/pnas.1013805107 PMC2993333 21041659

[59] Khalaf K Hana D Chou JT Singh C Mackiewicz A Kaczmarek M. Aspects of the tumor microenvironment involved in immune resistance and drug resistance. Front Immunol (2021) 12:656364. doi: 10.3389/fimmu.2021.656364 34122412 PMC8190405

[60] Wang J Zhang G Wang J Wang L Huang X Cheng Y. The role of cancer-associated fibroblasts in esophageal cancer. J Transl Med (2016) 14:30. doi: 10.1186/s12967-016-0788-x 26822225 PMC4732002

[61] Lv M Wang K Huang XJ. Myeloid-derived suppressor cells in hematological Malignancies: friends or foes. J Hematol Oncol (2019) 12(1):105. doi: 10.1186/s13045-019-0797-3 31640764 PMC6805310

[62] Li R Huang B Tian H Sun Z. Immune evasion in esophageal squamous cell cancer: From the perspective of tumor microenvironment. Front Oncol (2022) 12:1096717. doi: 10.3389/fonc.2022.1096717 36698392 PMC9868934

[63] Li Z Sun C Qin Z. Metabolic reprogramming of cancer-associated fibroblasts and its effect on cancer cell reprogramming. Theranostics (2021) 11(17):8322–36. doi: 10.7150/thno.62378 PMC8343997 34373744

[64] Martinez-Outschoorn UE Lin Z Trimmer C Flomenberg N Wang C Pavlides S. Cancer cells metabolically "fertilize" the tumor microenvironment with hydrogen peroxide, driving the Warburg effect: implications for PET imaging of human tumors. Cell Cycle (2011) 10(15):2504–20. doi: 10.4161/cc.10.15.16585 PMC3180189 21778829

[65] Zhang H Xie C Yue J Jiang Z Zhou R Xie R. Cancer-associated fibroblasts mediated chemoresistance by a FOXO1/TGFbeta1 signaling loop in esophageal squamous cell carcinoma. Mol Carcinog (2017) 56(3):1150–63. doi: 10.1002/mc.22581 27769097

[66] Qiao Y Zhang C Li A Wang D Luo Z Ping Y. IL6 derived from cancer-associated fibroblasts promotes chemoresistance *via* CXCR7 in esophageal squamous cell carcinoma. Oncogene (2018) 37(7):873–83. doi: 10.1038/onc.2017.387 29059160

[67] Che Y Wang J Li Y Lu Z Huang J Sun S. Cisplatin-activated PAI-1 secretion in the cancer-associated fibroblasts with paracrine effects promoting esophageal squamous cell carcinoma progression and causing chemoresistance. Cell Death Dis (2018) 9(7):759. doi: 10.1038/s41419-018-0808-2 29988148 PMC6037765

[68] Zhang H Yue J Jiang Z Zhou R Xie R Xu Y. CAF-secreted CXCL1 conferred radioresistance by regulating DNA damage response in a ROS-dependent manner in esophageal squamous cell carcinoma. Cell Death Dis (2017) 8(5):e2790. doi: 10.1038/cddis.2017.180 28518141 PMC5520705

[69] Winkler L Dimitrova N. A mechanistic view of long noncoding RNAs in cancer. Wiley Interdiscip Rev RNA (2022) 13(3):e1699. doi: 10.1002/wrna.1699 34668345 PMC9016092

[70] Papaspyropoulos A Lagopati N Mourkioti I Angelopoulou A Kyriazis S Liontos M. Regulatory and functional involvement of long non-coding RNAs in DNA double-strand break repair mechanisms. Cells (2021) 10(6):1506. doi: 10.3390/cells10061506 34203749 PMC8232683

[71] Zhang H Hua Y Jiang Z Yue J Shi M Zhen X. Cancer-associated fibroblast-promoted lncRNA DNM3OS confers radioresistance by regulating DNA damage response in esophageal squamous cell carcinoma. Clin Cancer Res (2019) 25(6):1989–2000. doi: 10.1158/1078-0432.CCR-18-0773 30463848

[72] Cheng Y Wang K Ma W Zhang X Song Y Wang J. Cancer-associated fibroblasts are associated with poor prognosis in esophageal squamous cell carcinoma after surgery. Int J Clin Exp Med (2015) 8(2):1896–903. PMC4402765 25932118

[73] Lee KW Sung CO Kim JH Kang M Yoo HY Kim HH. CD10 expression is enhanced by Twist1 and associated with poor prognosis in esophageal squamous cell carcinoma with facilitating tumorigenicity *in vitro* and *in vivo* . Int J Cancer (2015) 136(2):310–21. doi: 10.1002/ijc.29006 24895167

[74] Fu L Zhang C Zhang LY Dong SS Lu LH Chen J. Wnt2 secreted by tumour fibroblasts promotes tumour progression in oesophageal cancer by activation of the Wnt/beta-catenin signalling pathway. Gut (2011) 60(12):1635–43. doi: 10.1136/gut.2011.241638 21672941

[75] Ozawa D Yokobori T Sohda M Sakai M Hara K Honjo H. TGFBI expression in cancer stromal cells is associated with poor prognosis and hematogenous recurrence in esophageal squamous cell carcinoma. Ann Surg Oncol (2016) 23(1):282–9. doi: 10.1245/s10434-014-4259-4 25448803

[76] Kashima H Noma K Ohara T Kato T Katsura Y Komoto S. Cancer-associated fibroblasts (CAFs) promote the lymph node metastasis of esophageal squamous cell carcinoma. Int J Cancer (2019) 144(4):828–40. doi: 10.1002/ijc.31953 30367467

[77] Cai R Wang P Zhao X Lu X Deng R Wang X. LTBP1 promotes esophageal squamous cell carcinoma progression through epithelial-mesenchymal transition and cancer-associated fibroblasts transformation. J Transl Med (2020) 18(1):139. doi: 10.1186/s12967-020-02310-2 32216815 PMC7098101

[78] Yeo SY Ha SY Lee KW Cui Y Yang ZT Xuan YH. Twist1 is highly expressed in cancer-associated fibroblasts of esophageal squamous cell carcinoma with a prognostic significance. Oncotarget (2017) 8(39):65265–80. doi: 10.18632/oncotarget.17941 PMC5630329 29029429

[79] Ishibashi Y Tsujimoto H Einama T Mochizuki S Kouzu K Nomura S. Correlation between immunoinflammatory measures and periostin expression in esophageal squamous cell carcinoma: A single-center, retrospective cohort study. Ann Surg Oncol (2021) 28(2):1228–37. doi: 10.1245/s10434-020-08765-3 32613365

[80] Underwood TJ Hayden AL Derouet M Garcia E Noble F White MJ. Cancer-associated fibroblasts predict poor outcome and promote periostin-dependent invasion in oesophageal adenocarcinoma. J Pathol (2015) 235(3):466–77. doi: 10.1002/path.4467 PMC4312957 25345775

[81] Schoppmann SF Jesch B Riegler MF Maroske F Schwameis K Jomrich G. Podoplanin expressing cancer associated fibroblasts are associated with unfavourable prognosis in adenocarcinoma of the esophagus. Clin Exp Metastasis (2013) 30(4):441–6. doi: 10.1007/s10585-012-9549-2 23161183

[82] Santos AM Jung J Aziz N Kissil JL Pure E. Targeting fibroblast activation protein inhibits tumor stromagenesis and growth in mice. J Clin Invest (2009) 119(12):3613–25. doi: 10.1172/JCI38988 PMC2786791 19920354

[83] Zhen Z Tang W Wang M Zhou S Wang H Wu Z. Protein nanocage mediated fibroblast-activation protein targeted photoimmunotherapy to enhance cytotoxic T cell infiltration and tumor control. Nano Lett (2017) 17(2):862–9. doi: 10.1021/acs.nanolett.6b04150 28027646

[84] Duperret EK Trautz A Ammons D Perales-Puchalt A Wise MC Yan J. Alteration of the tumor stroma using a consensus DNA vaccine targeting fibroblast activation protein (FAP) synergizes with antitumor vaccine therapy in mice. Clin Cancer Res (2018) 24(5):1190–201. doi: 10.1158/1078-0432.CCR-17-2033 PMC5844837 29269377

[85] de Sostoa J Fajardo CA Moreno R Ramos MD Farrera-Sal M Alemany R. Targeting the tumor stroma with an oncolytic adenovirus secreting a fibroblast activation protein-targeted bispecific T-cell engager. J Immunother Cancer (2019) 7(1):19. doi: 10.1186/s40425-019-0505-4 30683154 PMC6347837

[86] Katsube R Noma K Ohara T Nishiwaki N Kobayashi T Komoto S. Fibroblast activation protein targeted near infrared photoimmunotherapy (NIR PIT) overcomes therapeutic resistance in human esophageal cancer. Sci Rep (2021) 11(1):1693. doi: 10.1038/s41598-021-81465-4 33462372 PMC7814141

[87] Watanabe S Noma K Ohara T Kashima H Sato H Kato T. Photoimmunotherapy for cancer-associated fibroblasts targeting fibroblast activation protein in human esophageal squamous cell carcinoma. Cancer Biol Ther (2019) 20(9):1234–48. doi: 10.1080/15384047.2019.1617566 PMC6741582 31185791

[88] Sato H Noma K Ohara T Kawasaki K Akai M Kobayashi T. Dual-targeted near-infrared photoimmunotherapy for esophageal cancer and cancer-associated fibroblasts in the tumor microenvironment. Sci Rep (2022) 12(1):20152. doi: 10.1038/s41598-022-24313-3 36418422 PMC9684531

[89] Gilardi L Airo FL Demirci E Clerici I Omodeo SE Ceci F. Imaging cancer-associated fibroblasts (CAFs) with FAPi PET. Biomedicines (2022) 10(3):523. doi: 10.3390/biomedicines10030523 35327325 PMC8945705

[90] Giesel FL Kratochwil C Schlittenhardt J Dendl K Eiber M Staudinger F. Head-to-head intra-individual comparison of biodistribution and tumor uptake of (68)Ga-FAPI and (18)F-FDG PET/CT in cancer patients. Eur J Nucl Med Mol Imaging (2021) 48(13):4377–85. doi: 10.1007/s00259-021-05307-1 PMC8566651 34137945

[91] Meyer C Dahlbom M Lindner T Vauclin S Mona C Slavik R. Radiation dosimetry and biodistribution of (68)Ga-FAPI-46 PET imaging in cancer patients. J Nucl Med (2020) 61(8):1171–7. doi: 10.2967/jnumed.119.236786 PMC7413240 31836685

[92] Rohrich M Syed M Liew DP Giesel FL Liermann J Choyke PL. (68)Ga-FAPI-PET/CT improves diagnostic staging and radiotherapy planning of adenoid cystic carcinomas - Imaging analysis and histological validation. Radiother Oncol (2021) 160:192–201. doi: 10.1016/j.radonc.2021.04.016 33940087 PMC9913884

[93] Su S Chen J Yao H Liu J Yu S Lao L. CD10(+)GPR77(+) cancer-associated fibroblasts promote cancer formation and chemoresistance by sustaining cancer stemness. Cell (2018) 172(4):841–856.e16. doi: 10.1016/j.cell.2018.01.009 29395328

[94] Chiu KJ Chiou HC Huang CH Lu PC Kuo HR Wang JW. Natural compounds targeting cancer-associated fibroblasts against digestive system tumor progression: therapeutic insights. Biomedicines (2022) 10(3):713. doi: 10.3390/biomedicines10030713 35327514 PMC8945097

[95] Puram SV Tirosh I Parikh AS Patel AP Yizhak K Gillespie S. Single-cell transcriptomic analysis of primary and metastatic tumor ecosystems in head and neck cancer. Cell (2017) 171(7):1611–1624.e24. doi: 10.1016/j.cell.2017.10.044 29198524 PMC5878932

[96] Tirosh I Izar B Prakadan SM Wadsworth MN Treacy D Trombetta JJ. Dissecting the multicellular ecosystem of metastatic melanoma by single-cell RNA-seq. Science (2016) 352(6282):189–96. doi: 10.1126/science.aad0501 PMC4944528 27124452

[97] Waise S Parker R Rose-Zerilli M Layfield DM Wood O West J. An optimised tissue disaggregation and data processing pipeline for characterising fibroblast phenotypes using single-cell RNA sequencing. Sci Rep (2019) 9(1):9580. doi: 10.1038/s41598-019-45842-4 31270426 PMC6610623

[98] Sebastian A Hum NR Martin KA Gilmore SF Peran I Byers SW. Single-cell transcriptomic analysis of tumor-derived fibroblasts and normal tissue-resident fibroblasts reveals fibroblast heterogeneity in breast cancer. Cancers (Basel) (2020) 12(5):1307. doi: 10.3390/cancers12051307 32455670 PMC7281266

[99] Chen K Wang Q Li M Guo H Liu W Wang F. Single-cell RNA-seq reveals dynamic change in tumor microenvironment during pancreatic ductal adenocarcinoma Malignant progression. EBioMedicine (2021) 66:103315. doi: 10.1016/j.ebiom.2021.103315 33819739 PMC8047497

[100] Li X Sun Z Peng G Xiao Y Guo J Wu B. Single-cell RNA sequencing reveals a pro-invasive cancer-associated fibroblast subgroup associated with poor clinical outcomes in patients with gastric cancer. Theranostics (2022) 12(2):620–38. doi: 10.7150/thno.60540 PMC8692898 34976204

[101] Zhang X Peng L Luo Y Zhang S Pu Y Chen Y. Dissecting esophageal squamous-cell carcinoma ecosystem by single-cell transcriptomic analysis. Nat Commun (2021) 12(1):5291. doi: 10.1038/s41467-021-25539-x 34489433 PMC8421382

[102] Dijkgraaf EM Santegoets SJ Reyners AK Goedemans R Wouters MC Kenter GG. A phase I trial combining carboplatin/doxorubicin with tocilizumab, an anti-IL-6R monoclonal antibody, and interferon-alpha2b in patients with recurrent epithelial ovarian cancer. Ann Oncol (2015) 26(10):2141–9. doi: 10.1093/annonc/mdv309 26216383

[103] Johnson DE O'Keefe RA Grandis JR. Targeting the IL-6/JAK/STAT3 signalling axis in cancer. Nat Rev Clin Oncol (2018) 15(4):234–48. doi: 10.1038/nrclinonc.2018.8 PMC5858971 29405201

[104] Patel RA Forinash KD Pireddu R Sun Y Sun N Martin MP. RKI-1447 is a potent inhibitor of the Rho-associated ROCK kinases with anti-invasive and antitumor activities in breast cancer. Cancer Res (2012) 72(19):5025–34. doi: 10.1158/0008-5472.CAN-12-0954 PMC3463757 22846914

[105] Hong D Kurzrock R Kim Y Woessner R Younes A Nemunaitis J. AZD9150, a next-generation antisense oligonucleotide inhibitor of STAT3 with early evidence of clinical activity in lymphoma and lung cancer. Sci Transl Med (2015) 7(314):314ra185. doi: 10.1126/scitranslmed.aac5272 PMC5279222 26582900

[106] Chen Y McAndrews KM Kalluri R. Clinical and therapeutic relevance of cancer-associated fibroblasts. Nat Rev Clin Oncol (2021) 18(12):792–804. doi: 10.1038/s41571-021-00546-5 34489603 PMC8791784

[107] Feig C Jones JO Kraman M Wells RJ Deonarine A Chan DS. Targeting CXCL12 from FAP-expressing carcinoma-associated fibroblasts synergizes with anti-PD-L1 immunotherapy in pancreatic cancer. Proc Natl Acad Sci USA (2013) 110(50):20212–7. doi: 10.1073/pnas.1320318110 PMC3864274 24277834

